# New Challenges and Opportunities: Extracellular Vesicles in Biological and Biochemical Processes

**DOI:** 10.3390/ijms26073395

**Published:** 2025-04-04

**Authors:** Ilaria Giusti, Celeste Caruso Bavisotto

**Affiliations:** 1 Department of Life, Health and Environmental Sciences, University of L’Aquila, 67100 L’Aquila, Italy; 2 Department of Biomedicine, Neurosciences and Advanced Diagnostic (BiND), Human Anatomy Section, University of Palermo, 90127 Palermo, Italy; celeste.carusobavisotto@unipa.it

## 1. Introduction

Cell-to-cell communication plays a crucial role in many processes, both in physiological and pathological assets [1,2]; this inter-cellular communication can be sustained by several mechanisms based on direct contact (e.g., electrical coupling, passage through gap junctions) or indirect interactions relying on the release of bioactive molecules, whether they are soluble or Extracellular Vesicle-associated.

Extracellular Vesicles (EVs) are membrane-enclosed structures released by all cells, and are unable to replicate on their own [3]. They range in size from a few nanometers to a little more than a micrometer and can be classified, rather than according to their size, according to their biogenesis. From this point of view, three main subtypes are considered: microvesicles (MVs) or ectosomes, exosomes (EXOs), and apoptotic bodies (ApoBs or ABs) [4,5,6]. MVs originate from an outward budding of the plasma membrane, which relies on several steps, such as cytoskeleton disassembly and the redistribution of plasma membrane lipids. EXOs originate from the endosomal compartment; an inward budding of the plasma membrane originates early endosomes. A further inward budding of their membrane originates some “intraluminal vesicles (ILVs)” inside a multivesicular body (MVB), and the following MVB fusion with the plasma membrane releases, into the extracellular space, the ILVs (at that point referred to as EXOs). ApoBs originate from cells undergoing apoptosis [5].

Although this classification remains valid, it is challenging to identify from an operational point of view what type of EVs has been isolated (there are no specific markers for specific subpopulations). However, it is possible to distinguish them according to their size. Even if there is no strict consensus on the following cut-offs, small EVs (sEVs) are usually defined as smaller than 200 nm in diameter, while they are classified as medium and large-EVs (mEVs, lEVs) if they are bigger than that [3,5].

Once released by the parental cell, the EVs interact with the target cells. They can be taken up by the target cell via caveolin-, lipid raft-, or clathrin-mediated endocytosis, phagocytosis, micropinocytosis, or direct membrane fusion, thus releasing the EV cargo into the recipient cells, or they can even bind to target cell surface receptors (the binding can be followed by fusion with the plasma membrane —receptor-mediated endocytosis—or by an intracellular signaling pathway activation) [7,8,9,10,11].

EVs contain a complex cargo composed of proteins, lipids, and nucleic acids, which can be fully functional once transferred to target cells. The consequence thereof is that EVs act as critical modulators in both physiological and pathological conditions; among the latter, cancer is certainly one of the most studied for two main reasons:

1. EVs are important mediators of communication in the tumor microenvironment [5,7,12,13,14,15,16]. Here, indeed, cell-to-cell EV-mediated communication sustains an active crosstalk between tumor and stromal cells [12,17] that modulates a plethora of processes involved in tumor development and progression: immune escape, angiogenesis, invasion, migration, fibroblast activation into CAFs (Cancer-Associated Fibroblasts), drug resistance, pre-metastatic niche formation, and metastasis spreading [18,19,20,21,22].
2. Since EVs reflect the composition of parental cells [5,23], they can be potentially very useful as biomarkers [23,24,25,26,27].

## 2. An Overview of the Published Articles

In this Special Issue, the focus was on the novel biochemical and molecular aspects of EVs in their role as communication mediators in biological processes, ranging from physiological to pathological conditions. Among the latter, cancer was certainly considered and, indeed, several papers (four out of nine) focused on this topic.

The reviews by Giusti, I. et al. and Burko et al. are certainly pertinent to this context. The first delves into the role of EVs in tumor metastasis and provides a general overview of the metastasis-related processes in which EVs are involved, considering their role in pre-metastatic niche formation and metastatic dissemination, as well as the possibility of their use in a liquid biopsy approach as biomarkers of metastatic disease (contribution 1). The second provides a comprehensive overview of the adaptation mechanisms underlying glioblastoma multiforme (GBM) resistance to radiotherapy, emphasizing the multifaceted nature of this process. The authors highlight that radioresistance arises from the tumor’s ability to adapt to radiation-induced stress through multiple interconnected factors, including cancer stem cells (CSCs), tumor heterogeneity, and microenvironmental influences such as hypoxia and metabolic reprogramming. Notably, the chaperone system plays a key role in maintaining proteostasis under radiation stress, while miRNAs and gene regulatory networks contribute to modulating the tumor’s adaptive response [20,28,29]. Furthermore, the study underscores the importance of DNA repair pathways in counteracting radiation-induced damage, ultimately sustaining tumor cell survival and proliferation. The discussion on EVs is particularly compelling, as they are implicated in the intercellular transfer of the oncogenic factors that enhance GBM resilience. Given their role in tumor adaptation and their potential as therapeutic vectors, EVs emerge as promising tools for overcoming GBM radioresistance and improving treatment outcomes (contribution 2).

As well as conducting reviews, some authors delved more into EV evaluation for peculiar clinical-related purposes in tumors. For example, Im et al. propose a study suggesting the potential use of miR-21 in EVs isolated from cerebrospinal fluid as a prognostic and therapeutic target for overcoming Metroxate (MTX) resistance in patients with leptomeningeal metastasis from non-small cell lung cancer (NSCLC-LM). First of all, knowing that high levels of miR-21 are found in the cerebrospinal fluid when these patients are subjected to MTX chemotherapy, the authors wanted to verify if the levels of miR-21 were indeed so unique and characteristic; therefore, they analyzed the cerebrospinal fluid miRNA profiles of seven patients compared to three healthy subjects and confirmed that miR-21 is the most highly expressed. Furthermore, they also confirmed that higher levels of miR-21 correspond to lower overall survival and are potentially involved in mediating MTX drug resistance. Subsequently, by using cell lines with different sensitivity towards MTX, they highlighted how the expression of miR-21 shows a very strong negative correlation with the sensitivity of the cells to MTX, indicating that miR-21 could negatively impact sensitivity towards this chemotherapy drug (i.e., decrease the MTX sensitivity). Knowing from the literature that miR-21 in the cerebrospinal fluid of these patients is mainly contained within the EVs, the authors demonstrated in vitro that EVs can transfer miR-21 to the recipient target cells, and then proceeded to determine whether the transport of miR-21 could influence cell proliferation and sensitivity to MTX, highlighting EVs’ ability to stimulate cell proliferation as well as MTX resistance. The same effects were supported by EVs isolated from the cerebrospinal fluid of patients with NSCLC leptomeningeal metastasis. Overall, these results suggest that miR-21, transferred from cell to cell via EVs, is potentially able to modulate MTX sensitivity (contribution 3).

Likewise, to be able to use EVs for potential diagnostic and prognostic purposes, Ueda et al. generated xenografts derived from female patients with uterine corpus malignancies. They evaluated several aspects of these interesting experimental models, including the release of EVs and their RNA profile. In general, the authors highlight how the generated xenografts retained several features and the gene profiles of primary tumors. More specifically, regarding EVs, the data show that the RNA profiles of EVs isolated from the primary tumor and EVs isolated from the corresponding xenograft are similar, suggesting that these models may serve as preclinical, tailored, customized research models for developing personalized medicine (contribution 4).

The studies published in this Special Issue, however, are not limited to processes related to tumor biology.

For example, one paper was concerned with the physiological role of EVs during embryonic development, contributing to the broadening of knowledge on EVs’ role in the communication between embryo and mother, which has recently been extensively reviewed in other works [30]. In that study by Muhandiram et al., to evaluate the role of EVs in embryo–maternal crosstalk during peri-implantation, the EVs released from trophoblast-mimicking cells were administered to endometrial-mimicking cells. Then, the secretomes of target cells were assessed, revealing that a 24 h EV treatment was able to induce a specific secretory response leading to a conditioned medium enriched with the proteins involved in growth, the cell’s ability to respond to external stimuli, antioxidant pathway activation, and cell junction; these proteins are known to be involved in embryo development and implantation. Notably, these effects were specifically induced by trophoblast EVs, since control EVs (from embryonic kidney cells) were not able to stimulate a similar secretion. Thus, the authors can conclude that trophoblast-derived EVs can modulate endometrial epithelial cell secretomes to ensure that they express the necessary proteins for facilitating the embryo implantation process (contribution 5).

In their paper, Darbinian et al. wondered if some fetal brain-derived exosome features could relate to the development of morphological abnormalities caused by prenatal alcohol exposure. It is known, indeed, that prenatal alcohol exposure can cause “fetal alcohol spectrum disorders (FASDs)”, which encompass neurodevelopmental disorders, physical birth defects (facial dysmorphology), or poor growth, just to name a few [31]. Firstly, the authors confirmed a series of damages caused in the fetus by alcohol exposure, such as eye development inhibition, the altered expression of some protein markers during brain/eye development, altered functions of neurons and astrocytes, and altered mRNA/protein expression in the eye/brain. Then, via immunoprecipitation, by contactin-2/TAG1 protein, they isolated fetal brain-derived exosomes from the blood of 10 mothers who used alcohol and compared them with control exosomes. In the first group, they highlighted a correspondence between some exosomal markers (mainly Myelin Basic Protein (MBP), but also others, such as synapsin-2 and fetal eye diameter. The assay was repeated on a larger group (120 fetuses, 60 controls, and 60 alcohol-exposed fetuses), where it was noticed that exosomal MBP levels significantly decreased in alcohol-exposed fetuses and that their eye size was consistently smaller. Their data suggest that exosomal MBP levels in mothers’ blood could serve as non-invasive markers to predict whether the child will be born with FASD-related developmental abnormalities, which cannot be detected with the available imaging methods (Ultrasounds or Magnetic Resonance Imaging) in the early phases of pregnancy (contribution 6).

Just as the role of EVs during embryo morphogenesis is known [32,33] (justifying their study as biomarkers of processes related to embryonic development, as in contribution 6), their involvement in immune-system related processes is equally well known [34,35,36], and Sokolov et al. were interested in this very aspect. Indeed, they explored lEV (large EV)-mediated communication between natural killer (NK) cells and monocytes during inflammatory processes and demonstrated lEVs’ ability to function as additional mechanisms involved in the immunomodulatory activity of NK cells on monocytes. In fact, they highlighted that EVs can negatively impact the viability of monocyte-like cells at certain concentrations (200 μg/mL). Lower doses of lEVs, when isolated from TNFα-activated NK cells (TNFα simulates the inflammatory activation of NK cells), were able to increase the phagocytic activity of monocytes, as well as the intensity of oxidative burst, compared to untreated monocytes. The authors, therefore, observed that the expression levels of some markers (such as CD54, CD71, and CD206) typical of activated monocytes was increased after treatment with lEVs (contribution 7).

Another paper deals with a completely different issue (indirectly demonstrating how broad the fields of interest related to EVs can be), namely, the quality of stored platelets; indeed, the debate on which are the most suitable methods for preserving platelets is still very open [37,38]. Thus, Kolenc et al. focus on the effect of 7 days of storage on platelet EVs, which can be correlated with platelet activation, thus being useful in providing information about platelet sample quality. After measuring the number of platelets and white blood cells in platelet concentrates stored at 20–24 °C for 1, 3, and 7 days (not highlighting significant differences), they evaluated the percentage of activated platelets, discovering that their proportion in the samples increased over the observation period. In parallel with platelet activation, the EV concentration increases in a time-dependent manner during storage: as platelet activation increases over storage time, more EVs are released. Thus, they supply some useful information to consider whether EV concentration could be a useful parameter as part of the quality control of stored blood units, and also debate if future studies should consider the impact of these EVs on transfusion recipients (contribution 8).

Wang et al. briefly touched on the field of virology, a field that has been widely studied regarding EVs in recent years [39,40,41]. In their review, they presented their recent findings on the crosstalk between exosomes and autophagy, and their connection with enveloped viruses infection, highlighting how this interplay between exosomes and autophagy could impact the pathogenesis of virus infections and represents a potential antiviral strategy, thus fueling vaccine research on enveloped virus infection (contribution 9).

## 3. Conclusions

This compilation of articles focused on the biological and biochemical processes sustained by Extracellular Vesicles in physiological and pathological conditions, underscoring even more the ongoing and vibrant interest in this research area, as confirmed by the constant increase in studies published on this topic [42,43]. This plethora of studies, though, confronts us with the reality that the more knowledge we gain, the more questions arise, making this research field an ever-present challenge, in which methodological advancements and new knowledge continue side by side, continually supporting each other.
